# Coating Formation on Ti-6Al-4V Alloy by Micro Arc Oxidation in Molten Salt

**DOI:** 10.3390/ma11091611

**Published:** 2018-09-04

**Authors:** Alexander Sobolev, Israel Wolicki, Alexey Kossenko, Michael Zinigrad, Konstantin Borodianskiy

**Affiliations:** Zimin Advanced Materials Laboratory, Department of Chemical Engineering, Biotechnology and Materials, Ariel University, Ariel 40700, Israel; sobolev@ariel.ac.il (A.S.); imwolicki@gmail.com (I.W.); kossenkoa@ariel.ac.il (A.K.); zinigrad@ariel.ac.il (M.Z.)

**Keywords:** micro arc oxidation, titanium coating, titanium oxide, molten salt

## Abstract

Micro Arc Oxidation (MAO) is an electrochemical surface treatment process to produce oxide protective coatings on some metals. MAO is usually conducted in an aqueous electrolyte, which requires an intensive bath cooling and leads to the formation of a coating containing impurities that originate in the electrolyte. In the current work, we applied an alternative ceramic coating to the Ti-6Al-4V alloy using the MAO process in molten nitrate salt at a temperature of 280 °C. The obtained coating morphology, chemical and phase composition, and corrosion resistance were investigated and described. The obtained results showed that a coating of 2.5 µm was formed after 10 min of treatment, containing titanium oxide and titanium‒aluminum intermetallic phases. Morphological examination indicated that the coating is free of cracks and contains round, homogeneously distributed pores. Corrosion resistance testing indicated that the protective oxide coating on Ti alloy is 20 times more resistive than the untreated alloy.

## 1. Introduction

Micro arc oxidation (MAO) is one of the most promising methods for the surface treatment of metals and alloys, and has recently received wide acceptance from various branches of industry. MAO is generally used to produce multipurpose wear-, corrosion-, and heat-resistant dielectric and decorative coatings on valve metals, such as Al, Mg, Ti, Ta, Nb, Zr, and Be [1,2,3,4,5].

Currently, titanium is an appealing metal due to its high specific strength [6], corrosion resistance [7], and excellent biocompatibility [8]. The use of MAO favors adapting surface composition, crystallographic structure, and morphology to achieve a large functionality that cannot be provided by the parent metal. The versatility of coatings obtained by MAO on Ti alloys, together with the simplicity and low cost of this treatment method, stimulated numerous attempts to coat titanium by the MAO [9,10,11,12] approach for various applications, including tribological, biomedical, dielectric, and photovoltaic coatings. Ti-6Al-4V is the most widely used Ti alloy as it contains stabilizer elements for both α and β phases for good creep and strength, respectively.

The mechanism of the MAO process is based on the anodizing electrochemical reaction, which occurs on a metallic surface and is accompanied by microarc discharge to form an oxide ceramic surface layer with a particular morphology and phase composition [13,14].

The formation of the coating results in numerous difficulties that affect different factors of the layer quality. Among those factors are the chemical composition, the concentration and temperature of the electrolyte, the duration of the treatment process, the chemical composition and structure of the substrate, and the electrical parameters of the MAO process [15,16,17,18]. The following factors affecting MAO in an aqueous electrolyte are considered as undesirable: the necessity of forced cooling of the treatment bath, an increased current density, the thermal dissociation of the electrolyte, the formation of compounds in the ceramic coating, and the low growth rate. Those issues can be solved by replacing an aqueous electrolyte with molten salts. The application of molten salt as an electrolyte in the MAO process has been reported by us in an earlier work on Al alloy surface treatment [19,20].

In the present work, the formation of a ceramic coating on Ti-6Al-4V alloy in a mixture of molten nitrate salts by MAO process was obtained. The chemical and phase composition of the obtained coating as well as its morphology and corrosion resistance are investigated and illustrated.

## 2. Materials and Methods

### 2.1. MAO Process

Titanium alloy Ti-6Al-4V rectangular specimens (Scope Metals Group Ltd., Bne Ayish, Israel, chemical composition shown in Table 1) with a surface area of 0.2 dm^2^ were ground using abrasive papers grits #280, #400, #600, #1000, #2400, and #4000, respectively, and then subjected to ultrasonic cleaning in acetone. The surface roughness is maintained to R_a_ = 3 μm after polishing.

**Figure 1:**
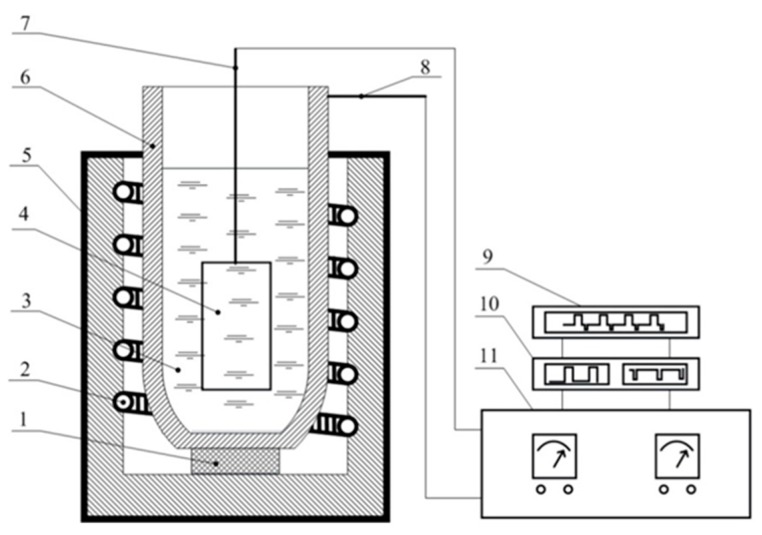
Schematic of experimental setup: 1—ceramic stand; 2—heating element; 3—molten salt electrolyte; 4—specimen subjected to MAO treatment; 5—furnace with automatic temperature controller; 6—nickel crucible; 7,8—current connectors; 9—data logger; 10—pulse generator; 11—power supply.

MAO treatment was performed at 280 °C in the electrolyte with a eutectic composition of KNO_3_-NaNO_3_ (Sigma-Aldrich, St. Louis, MO, USA) with the mass % of 54.3 and 45.7, respectively. The electrolyte was held in a nickel crucible (99.95% Ni), which served as a counter-electrode. The surface ratio of anode-to-cathode was 1:30, the anodic current density was 250 mA/cm^2^, and the voltage was limited by the galvanostatic mode. The applied power supply had the following parameters: I_max_ = 35 A, U_max_ = 1000 V; current and voltage were pulsed with a square-wave sweep at a frequency of 1 Hz (t_a_ = t_k_ = 0.5 s) by a Digit-EL PG-872 pulse generator (Minsk, Belarus). The duration of the MAO treatment was 10 min, with a coating growth rate of 0.25 µm/min. Finally, the obtained specimens were air-cooled, rinsed with distilled water, and dried. The behavior of current vs. time and voltage vs. time was recorded by a Fluke Scope Meter 199C (Eindhoven, The Netherlands) (200 MHz, 2.5 GS s^−1^). A schematic of the detailed experimental setup is given in Figure 1.

### 2.2. Characterization Techniques

The morphology examinations of the obtained coatings were done on the cross section of the treated specimen by TESCAN MAIA3 scanning electron microscopy (SEM) (Brno, Czech Republic) equipped with an energy dispersive X-ray spectroscopy (EDS) system by Oxford Instruments (Abingdon, UK) with an X-Max^N^ detector. The phase analysis of the coating was determined by the X’Pert Pro diffractometer (PANalytical B.V., Almelo, The Netherlands) with Cu_α_ radiation (λ = 1.542 Å) at the grazing incidence mode (angle of 3°) with a 2θ range from 30° to 80° (step size of 0.03°) at 40 kV and 40 mA.

The corrosion behavior of the treated and untreated specimens was examined by a potentiodynamic polarization test in a 3.5 wt % NaCl (Sigma-Aldrich Co.) solution by PARSTAT 4000A potentiostat/galvanostat (Princeton Applied Research, Oak Ridge, TN, USA). A three-electrode cell configuration was used for the corrosion test, wherein a Pt sheet acted as a counter-electrode and saturated Ag/AgCl (Metrohm Autolab B.V., Utrecht, The Netherlands) acted as a reference electrode. The polarization resistance of a sample was detected at the range of ±250 mV with respect to the recorded corrosion potential at a scan rate of 0.1 mV/s. Prior to the potentiodynamic polarization test, the samples were kept in the 3.5 wt % NaCl solution for 60 min in order to reach the steady state of a working electrode.

## 3. Results and Discussion

### 3.1. MAO Process Characterization

**Figure 2:**
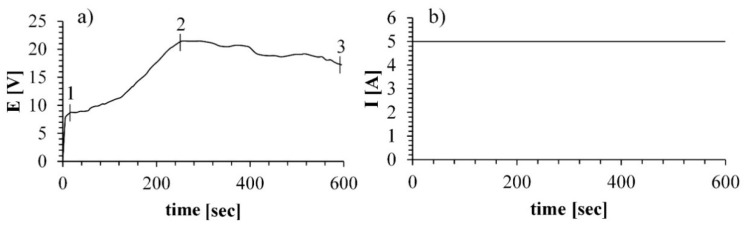
Plot of electric parameters of the MAO process applied on alloy Ti-6Al-4V: (**a**) voltage as a function of treatment time; (**b**) current as a function of treatment time.

Plots of voltage and current as a function of time during the MAO process are presented in Figure 2a,b, respectively.

Here, we sought to establish optimal conditions for plasma-mediated oxidation of Ti alloy. Briefly, the sample was immersed in a molten salt electrolyte in a nickel crucible, and the voltage was applied so that the sample served as a positive pole and the crucible as a negative pole. During the first few seconds of the process, a double electric layer was formed, followed by the charging, accompanied by the adsorption of gas bubbles and the formation of an amorphous film on the specimen surface (Figure 2a area 0–1). With the increase in the treatment time, a thicker oxide layer was formed, followed by a dielectric breakdown (Figure 2a area 1–2), which was accompanied by the formation of sparks on the specimen surface. It can be noted that during the MAO process, the voltage turns to the stationary mode after 300 s, meaning that the sparking process moves into the so-called micro arc oxidation mode (Figure 2a, area 2–3).

The process applied in molten salt is conducted at significantly lower potentials, about 22 V compared to the potentials of 300–600 V in the process conducted in aqueous electrolyte [21]. The current values of both processes are in the same range [22]. Those parameters indicate that the MAO process in molten salt is a more energy-efficient process and therefore is more economically beneficial.

### 3.2. Morphology and Elemental Analysis

**Figure 3:**
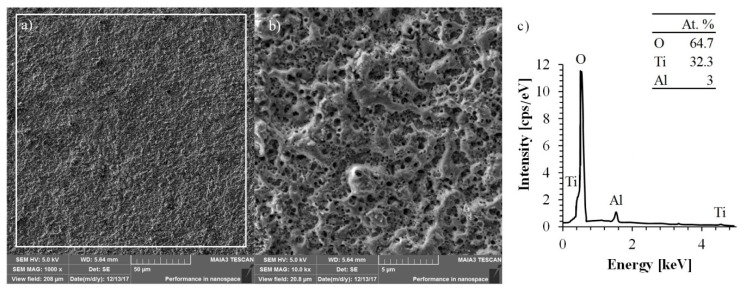
SEM image of surface morphology of the alloy Ti-6Al-4V treated by MAO with magnification: (**a**) 1000×; (**b**) 10,000× and (**c**) the elemental composition obtained by EDS.

The surface morphology and chemical compositions of the specimen treated by MAO process were investigated by SEM and EDS, respectively. The corresponding SEM and EDS images are shown in Figure 3a–c.

The surface of the oxide coating has a typical morphology usually obtained by the MAO process upon valve metals [23]. The homogeneously distributed round-shaped pores formed on the surface in the locations where the electrical high-temperature breakdowns took place.

During the electrical breakdown, the temperature of the discharge reached several thousand degrees, as noted by estimations made by Hussein et al. [24], resulting in the creation of a newly formed oxide layer that was first melted and then recrystallized. Surface morphology investigation showed that the formed coating has no cracks on the surface, indicating a low cooling rate of the newly formed oxides. Additionally, the obtained surface consists of round pores of a diameter ranging from 0.15 μm up to 0.5 μm. These pores are significantly smaller than the pores formed in the MAO process conducted in aqueous electrolyte (usually 2.5–15 μm, depending on the applied potential and processing time [25]).

The atomic composition of titanium, aluminum, and oxygen obtained by EDS analysis were 32.3%, 3.0%, and 64.7%, respectively. These values clearly indicate that the formed coating is free of impurities. That is contrary to the coating obtained by MAO treatment in aqueous electrolyte, which usually includes additional components originating from the electrolyte [26,27].

### 3.3. Phase Analysis

**Figure 4:**
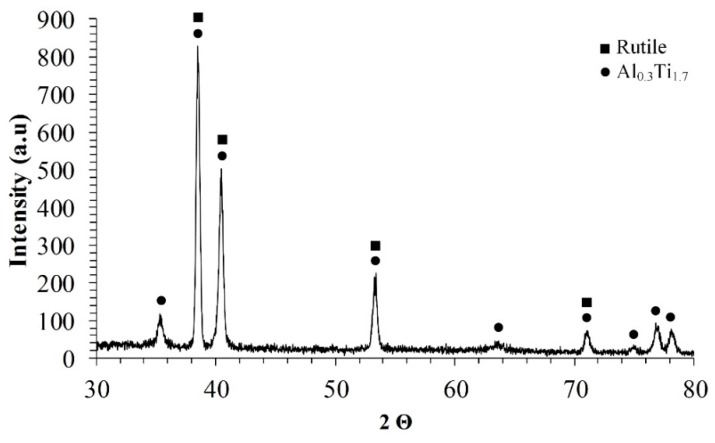
X-ray diffraction pattern of the alloy Ti-6Al-4V surfaces after MAO treatment.

The XRD pattern of the alloy Ti-6Al-4V surface after MAO treatment is shown in Figure 4.

XRD investigation evaluated the presence of the following phases in the obtained oxide-based coating: titanium dioxide in the form of rutile [28] and intermetallic of Al_0.3_Ti_1.7_ [29]. They were expected to be formed in the coating, rutile due to the oxidation process and Ti/Al intermetallic resulting in the noticeable presence of Al in the alloy. XRD measurements evaluated that no new phase was formed during the process and no impurities were detected in the coating.

**Figure 5:**
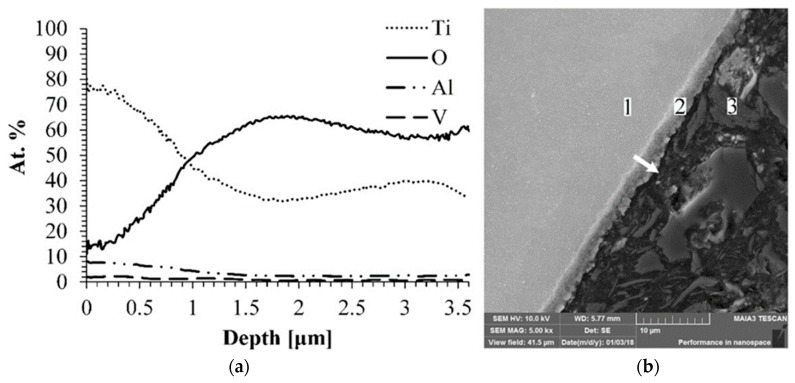
EDS line scan of the alloy Ti-6Al-4V after MAO treatment (**a**) and its cross section microphotograph obtained by SEM (**b**). Arrow indicates the direction of the EDS line scan analysis. Points in images attributed to: 1—base alloy; 2—oxide layer; 3—resin.

The cross section line scan and the microstructure of the Ti-6Al-4V alloy treated by MAO are shown in Figure 5.

SEM micrograph, jointly with the EDS line scan, indicated that the obtained oxide layer is uniform and its thickness is about 2.5 µm. Moreover, the elemental analysis detected only components that fit the composition of the expected oxide layer and no additional impurities. Usually, impurities are detected in the coating after the process that is carried out in the aqueous electrolyte. Aliasghari et al. detected the presence of phosphorous in the coating formed on Ti by the MAO process in an electrolyte containing phosphoric acid [30].

### 3.4. Corrosion Resistance Investigation

**Figure 6:**
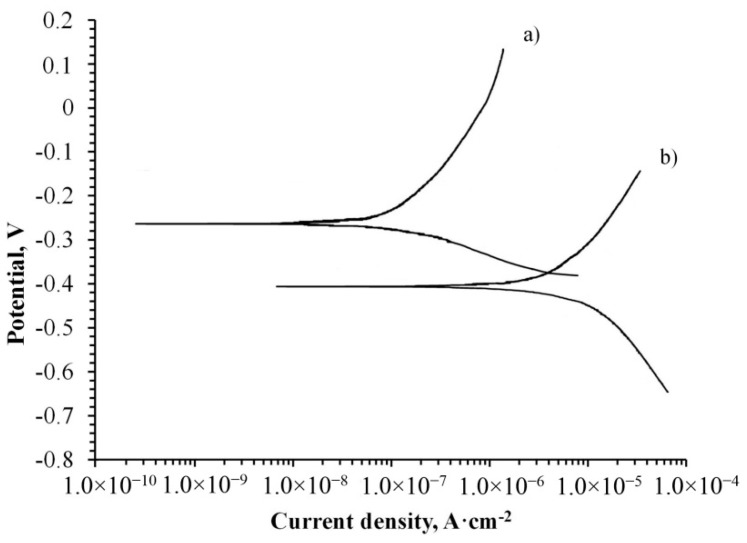
Potentiodynamic polarization curves for alloy Ti-6Al-4V (a) treated by MAO process and (b) untreated specimen. Both examined in 3.5 wt % NaCl.

The corrosion properties of the treated specimen were determined by the potentiodynamic polarization method. The obtained curve on the coated specimen was compared to the curve of the untreated and both are illustrated in Figure 6.

The obtained curves in Figure 6 are presented in semi-logarithmic coordinates. A higher corrosion resistance of the specimen is obtained when the corrosion potential is higher and the corrosion current density is lower. Therefore, it is clearly seen that the corrosion potential of the coated specimen shifted to be more positive and the current density to a more negative value, providing higher corrosion protection to the alloy. That may indicate the reduction of the anodic and cathodic processes due to the presence of a newly formed protective oxide layer on the metallic surface. The movement of the corrosion potential towards the anodic area also indicates the improvement of the treated specimen’s resistance.

Based on the corrosion currents and obtained slopes of cathodic and anodic curves, the polarization resistance (R_p_) was calculated according to Equation (1):

(1) $$\;R_{p}=\;\frac{\beta_{a}\times {\;\beta }_{c}}{2.3\times i_{\mathrm{corr}}\left(\beta_{a}+\beta_{c}\right)}$$

The Tafel slopes, β_a_ and β_c_, were calculated from the anodic and cathodic curves on the plot. Results of calculations that present the corrosion potentials (E_corr_), corrosion current densities (i_corr_), and the polarization resistance (R_p_) are summarized in Table 2.

The calculations presented in Table 2 show that the polarization resistance of the treated specimen is 213.76 kΩ/cm^2^, while the untreated specimen has a resistance of 10.98 kΩ/cm^2^. Those values show that the oxide protective coating on Ti alloy is almost 20 times higher than the untreated one. Our results, together with those of additional previous works [31,32], lead to the conclusion that MAO treatment can be applied to improve the corrosion resistance of metals.

The polarization resistance of an alloy treated in molten salt is higher than that of a similar alloy obtained in aqueous electrolyte [33]. This can be attributed to the lack of impurities in the coating and the presence of smaller pores, which conduct current and therefore reduce corrosion resistance.

## 4. Conclusions

A new approach to ceramic protective coating formation by the MAO process in molten salt was described. A TiAl6V4 alloy has been subjected to MAO treatment in a eutectic nitrate molten salt mixture. The thickness of the oxide coating reached 2.5 µm and the morphology examination evaluated the presence of a typical structure with homogeneously distributed round pores.

Phase composition analysis detected the presence of titanium oxide and titanium aluminum intermetallic (Al_0.3_Ti_1.7_). This was confirmed by EDS analysis.

The protective coating was subjected to corrosion resistance testing, and it was found that the specimen by coated MAO treatment is 20 times more resistant than the untreated specimen.

A comparison of the process conducted in molten salt with the process conducted in aqueous electrolyte showed the following benefits: the pores obtained on the surface are smaller, the coating has no impurities, the corrosion resistance is higher, and the process is economically beneficial due to the significantly lower potentials applied.

## Figures and Tables

**Table 1:** Chemical composition of the alloy Ti-6Al-4V.

Chemical Element, mass %			
V	Fe	Al	Ti
4	0.11	6	Base

**Table 2:** Calculated corrosion test results of untreated alloy Ti-6Al-4V and treated by MAO process specimens. Both examined in 3.5 wt % NaCl.

Samples	E_corr_ [mV]	i_corr_ × 10^−6^ [A]	β_a_ [mV/decade]	β_c_ [mV/decade]	R_p_ × 10^3^ [Ω/cm^2^]
Untreated alloy Ti-6Al-4V	−398	6.95	395	316	10.98
Treated alloy Ti-6Al-4V	−260	0.16	390	97	213.76
